# Role of the angiogenic components, VEGFA, FGF2, OPN and RHOC, in urothelial cell carcinoma of the urinary bladder

**DOI:** 10.3892/or.2012.1948

**Published:** 2012-08-03

**Authors:** APOSTOLOS ZARAVINOS, DIMITRIOS VOLANIS, GEORGE I. LAMBROU, DIMITRIS DELAKAS, DEMETRIOS A. SPANDIDOS

**Affiliations:** 1 Laboratory of Virology, Medical School, University of Crete, 71110 Heraklion, Crete; 2 Department of Urology, Asklipieio General Hospital, 16673 Voula, Athens; 3 First Department of Pediatrics, Choremeio Research Laboratory, University of Athens, 11527 Athens, Greece

**Keywords:** angiogenesis, VEGFA, FGF2, OPN, RHOC, urinary bladder

## Abstract

The objective of this study was to analyze the expression profile of the angiogenic components, vascular endothelial growth factor-A (VEGFA), basic fibroblast growth factor-2 (FGF2), osteopontin (OPN) and ras homolog gene family, member C (RHOC), in urothelial cell carcinoma (UCC) of the urinary bladder and to examine their role as candidate diagnostic biomarkers. Using qPCR, 77 samples of UCC of the urinary bladder and 77 matched tumor-associated normal samples were investigated to determine the expression of the four angiogenic components. The correlation between gene expression, patient survival and pathological features of the tumors was also examined. The VEGFA and OPN transcript levels were greater in the bladder cancer tissue than in the normal urothelium (P<0.001). Patients with higher VEGFA mRNA levels showed a tendency towards shorter cancer-specific survival. OPN levels showed a gradual increase, the lowest levels being found in non-invasive carcinoma and the highest in muscle invasive tumors. Elevated OPN levels indicated poor prognosis in connection with advanced disease stage (P<0.001). Both superficially invasive and muscle invasive tumors had significantly higher FGF2 levels compared to the control tissues (P=0.018 and P=0.050, respectively). Moreover, FGF2 was significantly higher in the metastatic vs. the non-metastatic tumors (P=0.0097). FGF2 levels exhibited a trend towards a correlation with worse patient survival. RHOC mRNA levels were higher in muscle invasive compared to superficially invasive tumors, as well as in grade III vs. grade I/II tumors. Furthermore, we detected worse overall survival for patients with high RHOC expression levels. VEGFA and FGF2 exhibited the best linear combination in the ROC curves for specificity and sensitivity. Thus, VEGFA and FGF2 may serve as candidate biomarkers for diagnostic purposes. Higher OPN expression may be used as a potential biomarker to predict patient survival relative to advanced tumor stage. However, further studies are required to investigate its role in urinary bladder carcinogenesis.

## Introduction

Angiogenesis plays a crucial role in the survival, proliferation and metastatic potential of several tumors, including urothelial cell carcinoma (UCC) of the urinary bladder (1). Clinically, the importance of the angiogenic response observed within a tumor should be considered as an independent prognostic factor for superficial as well as invasive bladder tumors (2). In early development, under hypoxic conditions, tumor neovascularization is observed by intensified microvessel density (MVD) and is regulated by hypoxia-inducible factor-1α (HIF-1α), whose overexpression induces the activation of the angiogenic growth factors, vascular endothelial growth factor-A (VEGFA) and basic fibroblast growth factor-2 (FGF2) (3).

VEGFA acts on endothelial cells and has various effects, including mediating increased vascular permeability, inducing angiogenesis, vasculogenesis and endothelial cell growth, promoting cell migration and inhibiting apoptosis. VEGFA and other angiogenic factors expressed under hypoxic conditions have been proposed as important tumor prediction factors in UCC (4,5).

During the early stages of tumor growth, FGF2 expression has been reported to play an important role in the regulation of angiogenesis, tumorigenicity and subsequent metastasis of human bladder cancer (6).

Osteopontin (OPN) plays a pivotal role in cell adhesion, chemotaxis, prevention of apoptosis, invasion, migration and anchorage-independent growth of tumor cells. Extensive research has demonstrated the pivotal role of OPN in the regulation of cellular signaling, which controls neoplastic and malignant transformation (7). Elevated osteopontin expression has also been found to be related to angiogenesis, tumor cell invasion and metastasis in UCC of various stages (8–10).

Ras homolog gene family, member C (RHOC) is essential for angiogenesis induced by VEGF, and is the downstream regulator of VEGF in endothelial cells (11). Its increased expression has also been linked to cell proliferation, increased invasion and metastasis in bladder cancer (12–14).

In the present study, we explored the expression of the angiogenic components, VEGFA, FGF2, OPN and RHOC, in UCC of the urinary bladder and investigated their potential role as diagnostic markers.

## Patients and methods

### Patients and tumor samples

A total of 77 paired samples, consisting of tumor and normal urothelium samples, were obtained from 77 Greek patients with UCC of the urinary bladder and examined to determine the expression of VEGFA, OPN, FGF2 and RHOC. Written informed consent in accordance with the Institutional Committee for the Protection of Human Subjects was obtained from all patients. Ethical approval of the study was obtained from the Institutional Review Board of the Asklipieio General Hospital and the University of Crete. All 77 patients were treated at the Asklipieio General Hospital, Voula, Athens. The patients included 68 males and 9 females; mean age, 72.12 years (men, 71.42 years and women, 74.44 years), ranging from 43 to 93 years (men, 44–93 years and women, 43–86 years). None of the 77 patients was previously administered systemic chemotherapy or external radiation therapy. Of the 77 patients, 56 had newly diagnosed bladder cancer and 21 had recurrent disease.

### Total RNA extraction, reverse-transcription, qPCR and clustering

Total RNA extraction and reverse-transcription were performed as previously described (15,16). The primers used for the amplification of the genes were as previously described (10,14). Amplification was performed using the GoTaq qPCR Master Mix (Promega, Madison, WI) and gene expression levels were analyzed on a Mx3000P thermal controller (Stratagene, La Jolla, CA). The thermal profile and the specifics of the qPCR used, were as previously reported (17).

### Enrichment and literature networks analysis

Gene Ontology (GO) enrichment evaluated each gene's biological process, molecular function and cellular component. Kyoto Encyclopedia of Genes and Genomes (KEGG) molecular pathway analysis was performed to identify possible enrichment of the genes. Further analysis was performed using the WikiPathways and Pathway Commons pathway enrichment methods. All enrichment analyses were performed using the WebGestalt web-tool (http://bioinfo.vanderbilt.edu/webgestalt). The BioNetwork Tools from www.pubgene.org was used to investigate the correlations among the angiogenic components.

### Statistical analysis

Differences in the expression levels between bladder cancer and adjacent normal tissue samples were evaluated using the Wilcoxon matched pairs test. The Kaplan-Meier method was used to estimate survival as a function of time and survival differences were assessed by the log-rank test. Numerical values in the scatterplots are expressed as the means ± SEM. Statistical significance was set at the 95% level (^*^P<0.05 and ^**^P<0.001). The cut-off scores for the analysis were obtained by receiver-operating characteristic (ROC) curve analysis and the area under curve (AUC) was utilized as a measure of the level of separation.

## Results

**Figure 1:**
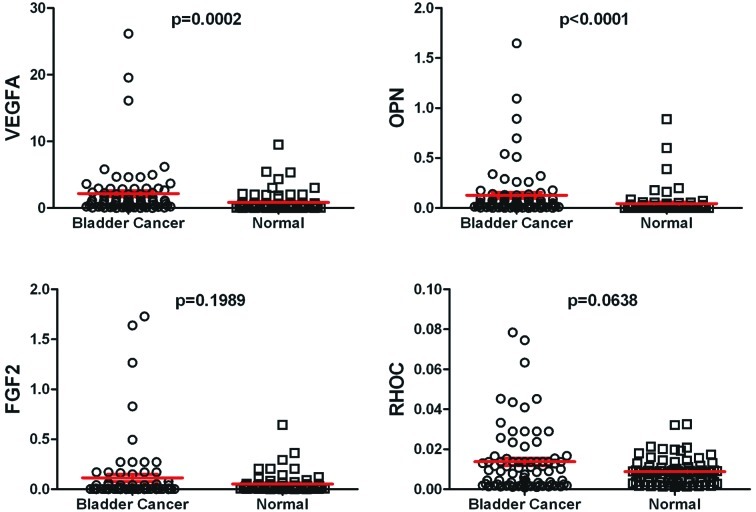
Scatterplots showing the mRNA expression levels of VEGFA, OPN, FGF2 and RHOC in the bladder cancer and normal urothelium samples. The bars indicate the mean ± SEM.

**Figure 2:**
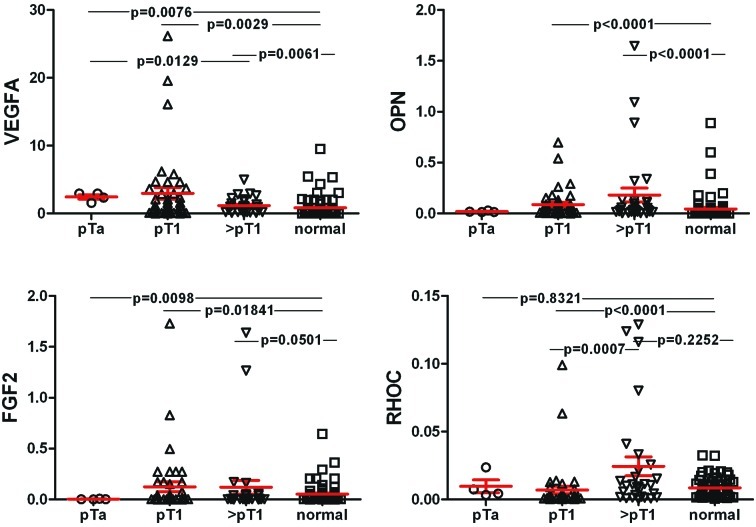
Scatterplots depicting the mRNA expression levels of VEGFA, OPN, FGF2 and RHOC in the non-muscle invasive, muscle invasive tumors and the controls. The bars indicate the mean ± SEM.

**Figure 3:**
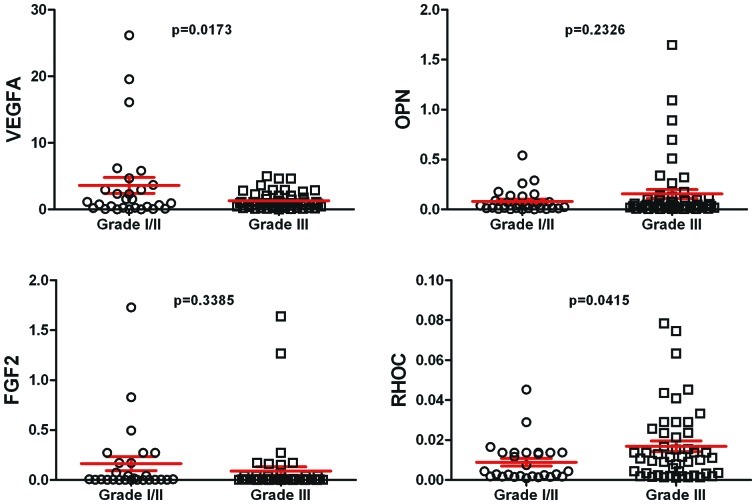
Scatterplots depicting the mRNA expression levels of VEGFA, OPN, FGF2 and RHOC in tumors of grades I/II and III. The bars indicate the mean ± SEM.

VEGFA and OPN mRNA levels were significantly higher in the UCC vs. the control tissue (P=0.0002 and P<0.0001, respectively). VEGFA and OPN levels were higher in the metastatic tumors compared to the non-metastatic ones, but the difference did not reach statistical significance. FGF2 levels did not exhibit a significant difference between the UCC and the control samples. Of note, the FGF2 levels were significantly higher in the metastatic vs. the non-metastatic bladder cancers (P=0.0097). RHOC levels tended to be higher in the tumors vs. the normal urothelia; however, this difference was marginally significant (P=0.0638) (Fig. 1). Moreover, RHOC levels were equal among metastatic and non-metastatic tumors (P=0.4140). The expression levels of the 4 angiogenic components were also investigated relative to the stage and grade of the tumors. VEGFA levels were significantly higher in all tumor stages vs. the normal tissue. OPN was higher in tumors of stage pT1 and those of >pT1 compared to the controls (P<0.0001). FGF2 levels were higher both in tumors of stage pT1 and those of >pT1 compared to the controls (P=0.018 and 0.05, respectively). RHOC levels were higher in tumors of stage >pT1 vs. those of pT1 (P=0.0007) (Fig. 2). VEGFA levels were higher in tumors of grade I/II vs. those of grade III (P=0.0173). OPN exhibited a trend for higher levels in grade III vs. grade I/II tumors; however, the difference was not significant. No difference was detected for FGF2 as regards the tumor grade, whereas RHOC levels were significantly higher in grade III tumors vs. those of grade I/II (P=0415) (Fig. 3).

**Figure 4:**
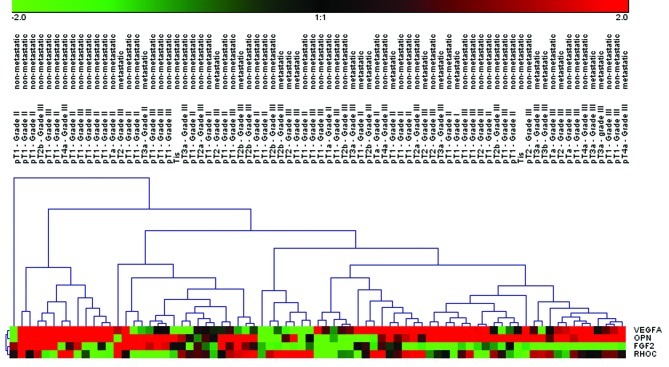
Two-way average-linkage hierarchical clustering (HCL) with Euclidian distance. Five big clusters can be noticed regarding the tumor samples and each cluster is further characterised by various sub-clusters. VEGFA with OPN (cluster 1) and FGF2 with RHOC (cluster 2) exhibited similar profiles and thus, are more closely clustered. Each row in the diagram represents a gene and the column the mean values of the fold-change in the gene expression between the tumor and normal samples. Red/green blocks represent signal increase/decrease, respectively. A differential expression of less or more than 2.0-fold was set as the threshold for the HCL analysis.

Two-way average-linkage hierarchical clustering (HCL) with Euclidian distance was also performed. A detailed view of the sample cluster dendrogram, regarding the tumor stage/grade, as well as the metastatic potential of each tumor, is displayed in Fig. 4.

The correlation among the randomly distributed expression levels was examined using the Spearman's correlation test. The degree of association between the normalized expression levels in the two groups (bladder cancer and normal tissue) is presented in Table I.

**Figure 5:**
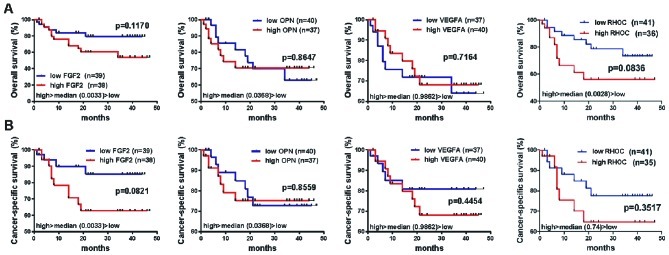
Kaplan-Meier curves depicting overall (A) and cancer-specific (B) survival (%) of the urinary bladder cancer patients, as regards the expression of the four angiogenic components (77 patients). Survival differences were assessed using the log-rank test. Statistical significance was set at the 95% level (P<0.05).

**Figure 6:**
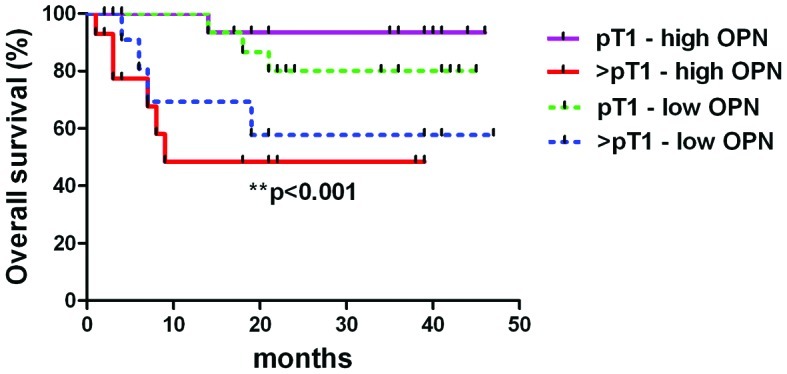
Kaplan-Meier curves depicting overall survival (%) of the urinary bladder cancer patients, as regards the expression of OPN, in association with the tumor stage. Muscle-invasive (>pT1) tumors correlated with worse overall patient survival, compared to high OPN levels in non-muscle invasive (pT1) tumors (P<0.001).

Analysis of disease-free and overall survival (77 cases) was evaluated in relation to the expression of the angiogenic components. The cases were divided into two groups with expression above (high expression levels) and below (low expression levels) the median expression of each gene. High FGF2 and RHOC levels exhibited a trend towards a correlation with worse patient survival (both overall and cancer-specific). Moreover, high VEGFA levels also tended to correlate with worse cancer-specific patient survival. Patients with deregulated OPN levels did not show any trend towards good or bad prognosis (Fig. 5). Of great interest, high OPN levels in patients with tumors of stage >pT1, correlated with worse overall survival, compared to high OPN levels in stage pT1 tumors (P<0.001) (Fig. 6).

**Figure 7:**
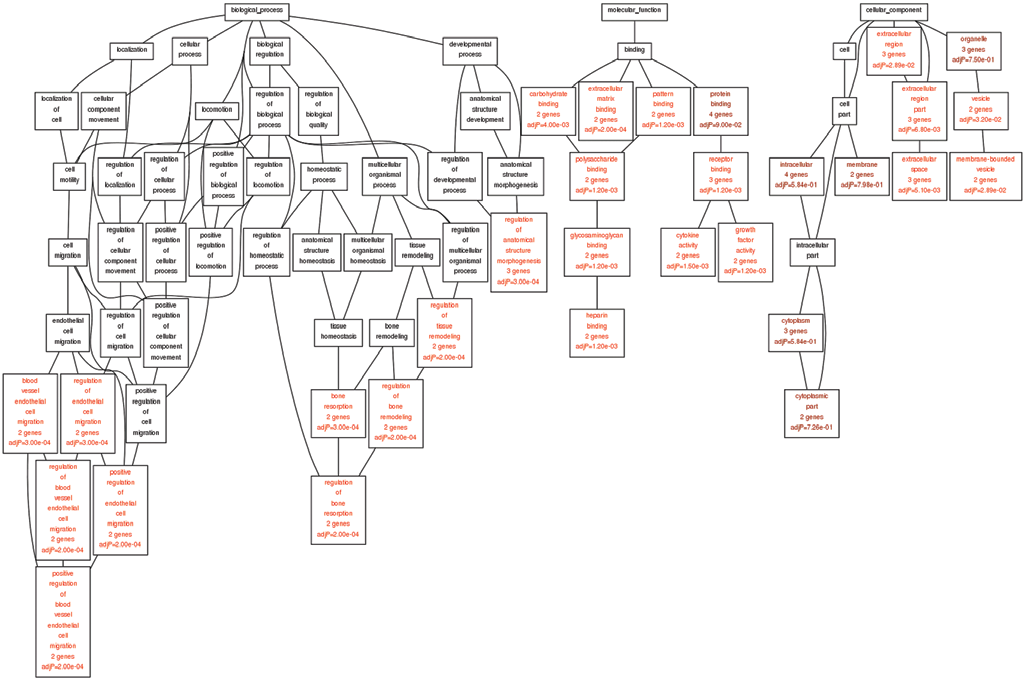
Gene Ontology (GO) diagram, depicting the most significant biological processes, molecular functions and cellular processes of VEGFA, OPN, FGF2 and RHOC.

GO analysis was performed in order to approach the functionality of the genes under study. As regards the biological processes of the genes, GO enrichment analysis revealed the regulation of endothelial cell migration and the regulation of tissue remodelling. Moreover, the angiogenic components had carbohydrate and extracellular binding properties, whereas they are components of the extracellular region. All the biological processes, molecular functions and cellular processes of the genes of interest, are depicted in Fig. 7 and Table II. KEGG enrichment analysis showed that VEGFA and OPN participate in the ‘focal adhesion’ pathway (adjP=0.0002) and VEGFA along with FGF2, both participate in ‘pathways in cancer’ (adjP=0.0003). WikiPathways enrichment analysis identified that VEGFA, FGF2 and OPN participate in ‘endochondral ossification’. Moreover, the Pathway Commons enrichment analysis showed that OPN, VEGFA and FGF2 are involved with ‘integrins in angiogenesis’ (adjP=4.08e-08), OPN and FGF2 participate in ‘proteogylcan syndecan-mediated signaling events’ (adjP=0.0001) and finally, VEGFA along with FGF2, play a role in the ‘glypican 1 network’ (adjP=0.0005) and in the ‘glypican pathway’ (adjP=0.0005).

**Figure 8:**
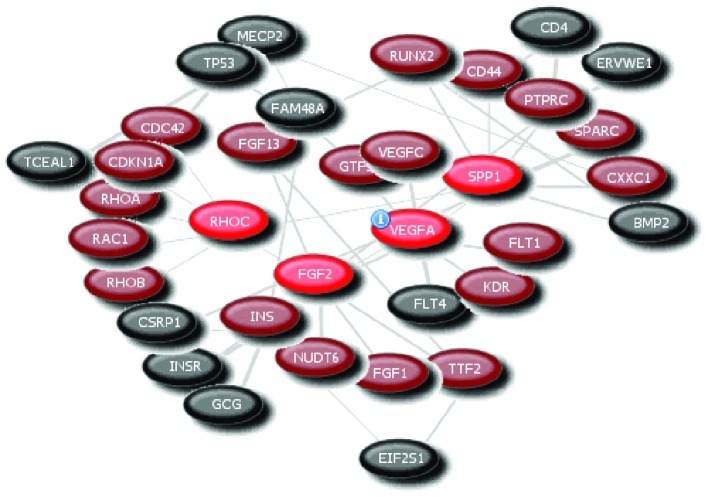
Networks viewed in the Network Browser show how multiple datasets containing gene names are co-cited in the literature, i.e., names that appear in the same text form a pair and are regarded as ‘neighbours’ in the network. When a gene is examined, its name is likely to appear in articles together with other gene names. The diagram shows how most genes under study are connected either directly or indirectly to each other in a Literature Network. Connections in the literature are a strong indicator of biological interaction.

Using the BioNetwork Tools from www.pubgene.org, correlations among VEGFA, FGF2, OPN and RHOC were further investigated. The results of our Literature Networks analysis are shown in Fig. 8.

**Figure 9:**
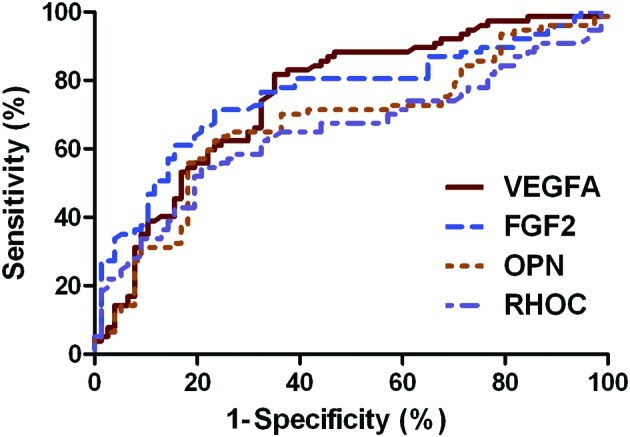
ROC curves used to evaluate the diagnostic performance of the angiogenic components. The area under curve (AUC) helps in the visualization of the trade-off between high sensitivity and high specificity when discriminating between bladder cancer and control tissue.

We also performed a ROC analysis to evaluate the diagnostic performance of the angiogenenic components. VEGFA was the most sensitive (81.8%) factor and FGF2 the most specific (76.6%) one, for distinguishing bladder cancer patients from the control samples, with an AUC of 0.751 (95% CI, 0.674–0.817) and 0.742 (95% CI, 0.664–0.810), respectively (Fig. 9). The results of all genes are shown in detail in Table III.

## Discussion

In this study, we analyzed the expression of the angiogenic components VEGFA, OPN, FGF2 and RHOC. The angiogenic factors VEGFA and FGF2 directly promote tumor angiogenesis. OPN supports tumor angiogenesis, invasion and metastasis and the GTPase RHOC plays a fundamental role in cytoskeleton-dependent processes including alterations in cell shape, polarity, adhesion, cell motility and cell to matrix interactions. We explored their expression values in 77 UCC and 77 matched histologically normal urothelium samples, and examined them in respect to the clinicopathological data of the patients.

VEGFA produces a number of important biological effects, such as endothelial mitogenesis and migration, extracellular matrix remodeling via the induction of proteinases, increased vascular permeability and maintenance of newly formed vasculature (18). Our results showed a significant overexpression of VEGFA in bladder cancer, verifying the results from previous reports (10,19). UCCs are characterized by markedly increased angiogenesis when compared with the normal urothelium from which they are derived. VEGFA is a crucial growth factor mediating tumor angiogenesis, and its expression has been associated with advanced grade, stage and recurrence of UCC (20). However, we did not detect any difference in the expression levels of VEGFA among tumors of stage pT1 and those of >pT1 and VEGFA expression was higher in grade I/II vs. grade III tumors. The prognostic importance of VEGFA in urothelial carcinoma has already been implicated in a several studies (4,5,21). In our study, substantial expression levels of VEGFA exhibited superior sensitivity with a good AUC score, and increased VEGFA expression levels existed even in superficial tumors (22) compared to the normal urothelium. Higher VEGFA levels were detected in tumors invading the lamina propria mucosae (pT1) and in muscle-invasive (pT2-T4) tumors compared to normal tissue. Moreover, high VEGFA levels tended to correlate with worse cancer-specific survival. VEGFA levels were higher in the metastatic tumors compared to the non-metastatic ones; however, the difference was not statistically significant. This could be attributed to the limited number of metastatic cases included in our study (n=20), compared to the non-metastatic ones (n=57), (P=0.0035; F test to compare variances). The VEGF pathway is emerging as an important therapeutic target for metastatic bladder cancer (23). Overall, there is substantial evidence to suggest that the essential role of VEGFA is to act as a diagnostic marker for the aggressiveness of UCC.

OPN expression has recently been suggested to play a role in angiogenesis, among multiple biological functions. OPN has been identified to play essential role in two key aspects of tumor progression: VEGF expression by tumor cells and VEGF stimulated neovascularization (24). In this study, we detected significantly increased OPN levels in bladder tumor tissue vs. the normal urothelium. High OPN expression levels in bladder cancer have recently been reported (10,19). OPN levels showed a gradual increase, the lowest levels being found in superficial carcinoma (22) and the highest values in muscle invasive (≥pT2) tumors. An increased OPN expression in superficially invasive (pT1) and muscle invasive (pT2) tumors contributes to tumor cell proliferation and metastasis. We showed that high OPN levels in bladder tumors of stage >pT1, correlated with worse overall patient survival, compared to high OPN levels in stage pT1 tumors. In this manner, elevated OPN levels indicated poor prognosis in connection to advanced disease stage. As in VEGFA, OPN levels were also higher in the metastatic tumors compared to the non-metastatic ones, although possibly due to the limited number of metastatic cases (n=20) this difference did not reach statistical significance.

During the early stages of tumor growth, FGF2 expression has been shown to play an important role in the regulation of angiogenesis, tumorigenicity and subsequent metastases of human bladder cancer (6). Significantly increased FGF2 protein levels have previously been reported in bladder cancer by a number of studies (22,25,26), and its expression has been positively correlated with tumor stage (27) and grade (26). FGF2 upregulation has recently been reported to be an independent predictor of clinical outcome in patients with UCC of the bladder (22). In the present study, FGF2 levels exhibited a trend to be higher in the UCC tissue compared to the normal urothelium, but the difference was not significant. However, both superficially invasive (pT1) and muscle invasive (pT2) tumors had significantly higher FGF2 mRNA expression compared to the control tissue. Moreover, no difference could be detected between tumors of grade I/II and those of grade III. Importantly, our results indicated that FGF2 expression was higher in the metastatic vs. the non-metastatic tumors. We also found that high FGF2 levels exhibited a trend towards a correlation with worse patient survival (both overall and cancer-specific) and in the ROC curve, FGF2 exhibited a very good specificity for distinguishing bladder cancer from the normal tissue.

RHOC has attracted attention as its increased expression has been linked to increased invasion and metastasis in bladder cancer (13). RHOC-induced cytoskeletal changes and the release of vascular permeability factors function cooperatively to mediate cell intravasation during the early stages of cancer cell metastasis (28). In the present study, we found marginally higher RHOC mRNA levels in the bladder cancer tissues compared to the normal tissues. Moreover, our results indicated higher RHOC mRNA levels for muscle invasive (pT2) vs. superficially invasive (pT1) tumors, as well as for grade III vs. grade I/II tumors. Previous reports have shown increased RHOC expression in UCC of the urinary bladder (13). Comparable patterns of expression have also been found in renal cell carcinoma (29) and ovarian carcinoma (30). In addition, high RHOC protein levels have been previously associated with poorer disease-free and overall survival in bladder cancer patients (13). In accordance with these studies, we detected a clear trend towards a worse overall survival for patients with high RHOC expression levels.

No significant correlation was observed between the expression levels of VEGFA, FGF2, OPN and RHOC in the bladder cancer tissue, with the exception of FGF2, which correlated with RHOC. This lack of correlation may be due to their roles in different steps of tumorigenesis, e.g., VEGFA overexpression supports early vascularisation under hypoxic conditions, whereas OPN is involved in advanced processes, such as tumor growth in deregulating the extracellular matrix.

In conclusion, our data confirm the involvement of high VEGFA and OPN levels in bladder cancer, as well as an important role for FGF2 and RHOC in this disease. In particular, there are convincing data suggesting that VEGFA may serve as a candidate biomarker for diagnostic purposes. Further studies are required to investigate the role of OPN in UCC of the urinary bladder.

## Figures and Tables

**Table I:** Spearman's correlations among VEGFA, OPN, FGF2 and RHOC in bladder cancer and normal tissue.

	Normal tissue			
	
Bladder cancer	FGF2	OPN	VEGFA	RHOC
FGF2		**0.567374**	**0.319375**	0.172785
OPN	0.096619		**0.434898**	−0.06099
VEGFA	−0.12037	0.069437		0.049927
RHOC	**0.231437**	0.131188	−0.21996	

The correlation co-efficient (CC) values with a P<0.05 are depicted in bold.

**Table II:** The most significant biological processes, molecular functions and cellular processes of VEGFA, OPN, FGF2 and RHOC are depicted after GO enrichment analysis.

Genes	Biological processes (adjP-value)	Molecular functions (adjP-value)	Cellular processes (adjP-value)
VEGFA, FGF2	Regulation of blood vessel endothelial cell migration (adjP=0.0002)	Growth factor activity (adjP=0.0012)	
	Positive regulation of blood vessel endothelial cell migration (adjP=0.0002)	Polysaccharide binding (adjP=0.0012)	
	Positive regulation of endothelial cell migration (adjP=0.0002)	Glycosaminoglycan binding (adjP=0.0012)	
	Blood vessel endothelial cell migration (adjP=0.0003)	Heparin binding (adjP=0.0012)	
	Regulation of endothelial cell migration (adjP=0.0003)	Pattern binding (adjP=0.0012)	
		Carbohydrate binding (adjP=0.0040)	
VEGFA, OPN	Regulation of bone remodeling (adjP=0.0002)	Extracellular matrix binding (adjP=0.0002)	Vesicle (adjP=0.0320)
	Regulation of tissue remodeling (adjP=0.0002) vesicle	Membrane-bounded (adjP=0.0289)	
	Regulation of bone resorption (adjP=0.0002)		
	Bone resorption (adjP=0.0003)	Cytokine activity (adjP=0.0015)	
VEGFA, FGF2, OPN	Regulation of anatomical structure morphogenesis (adjP=0.0003)	Receptor binding (adjP=0.0012)	Extracellular region part (adjP=0.0068)
			Extracellular region (adjP=0.0289)
			Extracellular space (adjP=0.0051)

**Table III:** ROC curve analysis.

	AUC	SE	95% CI	P-value	z statistic	Sensitivity^a^	Specificity^b^
VEGFA	0.751	0.0399	0.674–0.817	<0.0001	6.276	81.8	64.5
FGF2	0.742	0.0417	0.664–0.810	<0.0001	5.803	69.9	76.6
OPN	0.673	0.0442	0.586–0.759	<0.0001	3.839	64.9	74.0
RHOC	0.649	0.0451	0.560–0.737	0.0013	3.411	61.0	67.5

VEGFA was the most sensitive and FGF2 the most specific factor for distinguishing bladder cancer from the control samples.

a The fraction of those patients with the disease correctly identified as positive by the test.

b The fraction of those patients without the disease correctly identified as negative by the test.

## Abbreviations

UCC: urothelial cell carcinoma
qPCR: quantitative real-time polymerase chain reaction
HIF-1α: hypoxia-inducible factor-1α
VEGFA: vascular endothelial growth factor-A
FGF2: basic fibroblast growth factor-2
OPN: osteopontin
RHOC: ras homolog gene family, member C
HCL: hierarchical clustering
KEGG: Kyoto Encyclopedia of Genes and Genomes
ROC curve: receiver operating characteristic curve
AUC: area under the curve
